# Examining the Auditory Selective Attention Switch in a Child-Suited Virtual Reality Classroom Environment

**DOI:** 10.3390/ijerph192416569

**Published:** 2022-12-09

**Authors:** Carolin Breuer, Karin Loh, Larissa Leist, Stephan Fremerey, Alexander Raake, Maria Klatte, Janina Fels

**Affiliations:** 1 Institute for Hearing Technology and Acoustics, RWTH Aachen University, 52074 Aachen, Germany; 2 Cognitive and Developmental Psychology, University of Kaiserslautern-Landau, 67663 Kaiserslautern, Germany; 3 Audiovisual Technology Group, Technische Universität Ilmenau, 98693 Ilmenau, Germany

**Keywords:** auditory selective attention switch, binaural hearing, virtual reality, children

## Abstract

The ability to focus ones attention in different acoustical environments has been thoroughly investigated in the past. However, recent technological advancements have made it possible to perform laboratory experiments in a more realistic manner. In order to investigate close-to-real-life scenarios, a classroom was modeled in virtual reality (VR) and an established paradigm to investigate the auditory selective attention (ASA) switch was translated from an audio-only version into an audiovisual VR setting. The new paradigm was validated with adult participants in a listening experiment, and the results were compared to the previous version. Apart from expected effects such as switching costs and auditory congruency effects, which reflect the robustness of the overall paradigm, a difference in error rates between the audio-only and the VR group was found, suggesting enhanced attention in the new VR setting, which is consistent with recent studies. Overall, the results suggest that the presented VR paradigm can be used and further developed to investigate the voluntary auditory selective attention switch in a close-to-real-life classroom scenario.

## 1. Introduction

Auditory selective attention (ASA) describes the capability to focus one’s auditory attention on one sound source while suppressing other distracting sound sources. One popular effect in this field is the cocktail party problem, which was first described by Cherry [1]. Since then, the research on auditory selective attention has been ongoing, and yet, there are still mechanisms that need further investigation, such as the voluntary attention switch or age-related effects. In his experiments, Cherry gained the first insights into how dichotic speech can be filtered. By playing recorded speech dichotically and asking the participants to focus on one ear, he showed that unattended speech was successfully suppressed most of the time.

While current studies already create more realistic acoustic scenarios by using binaural sound reproduction, the next step is investigating the behavior of adults and children in more natural situations. A promising step towards creating those close-to-real-life settings is utilizing virtual reality (VR) technology. This allows researchers to bring real-life scenarios such as classrooms or offices into the laboratory and investigate not only complex acoustic environments, but also the audiovisual interactions such as the influence of complex acoustic or visual noise on cognitive processes. The current study, therefore, aimed to translate a previously validated, child-appropriate paradigm on auditory selective attention into a VR classroom. The proposed new scenario was then validated by comparing the results to the previous 2D version.

Before introducing the new paradigm in detail, a brief overview of auditory selective attention is provided, and current studies on the evaluation of cognitive tasks in virtual reality are discussed, showing some limitations and general remarks when translating established paradigms into VR.

### 1.1. Previous Experiments on Auditory Selective Attention

There are different theories regarding the unattended processing of speech signals. Broadbent’s filter theory suggests the processing of information at a low level, which assumes that signals are filtered according to basic features and that unattended speech is not processed semantically [2]. On the other hand, Deutsch and Deutsch [3] suggested a higher level of processing, where all information is examined semantically. Their theory states that the relevant information is selected only after the processing. Treisman [4] presented an intermediate theory called “attenuation theory”, which suggests that some unattended stimuli would be processed based on their intensity. Many studies have investigated these mechanisms using dichotic speech material, such as the experiments by Moray [5], as well as Wood [6], who found that around 30% of the participants would switch their attention to the unattended stream when being presented semantically meaningful and personally relevant information, i.e., their names. After switching to the unattended stream, participants remained focused on the unattended stream for a short period [6]. However, since these findings only hold for a small number of participants, the results are inconclusive regarding the underlying processing theories. A detailed review of the history of auditory selective attention research is given by Bronkhorst [7]. This is supplemented by an overview of the theories for explaining the ability to selectively attend to specific auditory stimuli by Murphy [8].

In contrast to the studies described, which all introduce an involuntary switching of auditory selective attention, a first version of the presented paradigm was developed by Koch et al. [9] to investigate the intentional switching of auditory selective attention. This first version used a dichotic presentation of spoken digits from one to nine, excluding five. In this scenario, two digits, spoken by a male and female voice, were presented simultaneously to the left and right ear. Before each trial, a visual cue indicated whether the participants should attend to the male or female voice. The task was to attend to the voice of the previously cued gender and classify whether the presented target digit was smaller or greater than five. To implement attention switches, the gender of the attended speaker was changed between trials. The results revealed significant switch costs, as well as congruency effects regarding the task-irrelevant stimuli, all in all suggesting the sluggishness of the auditory attention, which follows previous findings [7,9].

A limitation of the previously described paradigm was the dichotic reproduction, which was far from plausible. Therefore, a step toward more realistic listening scenarios was taken by Fels et al. [10] by adapting the paradigm including a binaural reproduction. This optimized paradigm was used, for example, by Oberem et al. [11,12,13] to investigate multiple influences on the intentional switching of ASA, such as different reproduction methods [11]. Next to real sound sources, binaural reproduction with cross-talk-cancellation (CTC) with loudspeakers, as well as binaural reproduction via headphones with individual and generic head-related transfer functions (HRTFs) were compared. Although the investigated main effects were observed in all reproduction methods, the irrelevant sound source could be filtered more easily when using the real sound sources or the individual HRTF [11]. These results suggested that an individual or generic binaural reproduction via headphones could be used for future studies and that a dynamic reproduction should be employed to further improve the binaural synthesis. In further steps, this paradigm has been extended to investigate the effects of reverberation time on auditory selective attention [12], as well as age effects in adults [13].

### 1.2. Auditory Selective Attention in Children

Another interesting aspect of auditory attention is the progression of the ability to focus one’s attention until adulthood. It has been shown that this skill develops in children with growing age. It is still discussed when the control over one auditory selective attention is fully developed. Some studies suggest the early teenage years [14]; others find adult-like performance around the age of 9–11 years when introducing tasks that involve the suppression of background noise [15]. Another study by Peng et al. [16] found evidence that the mechanisms to control ASA are not yet developed in elementary school children and suggested that a difference in inhibitory control could be the reason for more errors made by this age group. A review of the different stages of the development of the auditory system is given by Litovsky [17].

To investigate the switching of auditory selective attention in children, as well as the related developmental effects, the paradigm by Oberem et al. [12] was adapted for children by Loh et al. [18]. To be understandable for young children, the task was changed slightly. Instead of classifying numbers smaller or greater than five, the children’s task was to categorize animal names into groups of flying and non-flying animals. Among others, possible stimuli were “cat” as a non-flying animal or “owl” as a flying animal. A detailed description of the task, which was also applied in the current study, is given in Section 2.2 and Section 2.7 and . Next to the new stimuli, gamification elements such as visual feedback and a progress update were implemented. These modifications were made to keep the children motivated throughout the experiment. Other than the visual cue and the feedback elements presented on a computer display, no visual stimuli were used. The child-appropriate paradigm was validated with 24 children at the ages of 6–10 years, as well as 24 young adults in conditions with and without noise [18]. The first results indicated that children are more susceptible to noise than adults, which was reflected in higher error rates and lower reaction times. This suggests that children respond faster, but less precisely, when being in noisy conditions, which is referred to as a speed–accuracy trade-off. These results were in accordance with other literature [15,19]. The results further suggested that adults could benefit more from the spatial information provided by the binaural reproduction. Although even low noise levels were found to influence children’s auditory attention, the performance of children and adults regarding the voluntary attention switch was comparable [18]. These findings add to the previously discussed lack of understanding of the development of the auditory system in children. Loh et al. [18] therefore argued for the investigation of ASA in children below the age of six years.

### 1.3. Investigations of Cognitive Performance in VR

To create close-to-real-life situations and keep the participants engaged, game-like elements, as well as the use of VR have been established methods in cognitive research and training for many years now, with one of the first virtual classrooms having been presented over 20 years ago by Rizzo et al. [20]. In the past, many studies investigated the use of VR applications in psychological and cognitive training. One example is the training of teachers to improve their behavior in a classroom by giving a lecture in VR [21].

Furthermore, it is not clear if the direct transformation of paradigms, e.g., measuring cognitive load or attention in VR environments, is feasible. Even if studies investigate the use of VR for cognitive assessment, they rarely compare these results to a non-VR baseline [22]. Some studies suggest that the transformation of classical paradigms into VR is easily done [23]. For example, in the auditory domain, Schoeffler et al. [24] compared auditory quality ratings in both virtual and real settings and found only small differences between the environments. While this direct translation of the task and input methods may be feasible for some tasks, other factors such as the display size and test environment can influence the cognitive behavior and, therefore, the experimental outcome [25]. There are studies suggesting enhanced visual attention in VR applications using an HMD as opposed to using 2D setups or other displays, e.g., [22,26,27]. In a study on visual attention, Li et al. [26] used the same visual attention task on a 2D display and in an HMD-based VR environment, which is comparable to the present study, and evaluated EEG signals in both scenarios. By doing so, they found that visual attention was enhanced in VR. Furthermore, the performance of participants was better in VR as opposed to 2D, which was reflected in lower reaction times and error rates [26]. In accordance with that, Wan et al. [22] compared the impact of a 3D visualization presented on a conventional display or a VR version presented on an HMD using a game to measure working memory and attention. They found lower time to completion and higher game scores for the attention measure in VR mode than in 3D display mode. Another study by Makransky et al. [28] found that the presence was higher in VR, but learning was impaired, probably due to cognitive overload and distraction by the VR environment. It is important to note that not only the results regarding the enhanced presence and cognitive load caused by VR were inconclusive. Gamification in general can lead to more engagement of participants through enhanced enthusiasm and also increase the cognitive load. A systematic literature review is given by Lumsden et al. [29]. As stated through the VR examples, gamification does not have the same effects on all tasks; therefore, possible unknown influences on human cognition need to be considered during the development of new VR paradigms [22,26,30].

### 1.4. Investigating Children in VR

There are some drawbacks to bringing children and VR together, and great caution has to be taken in the design of the virtual environment. Many manufacturers of consumer-grade VR technology such as head-mounted displays (HMDs) have an age restriction for children below the age of 13 [31]. Furthermore, many HMDs are too big for children, since children’s heads and, therefore, the distances between the eyes are smaller than adults’. Studies suggest that also pupil distance increases until early adulthood and reaches an adult-like width at the age of 16 to 25 years [32]. Still, there have been various studies investigating children’s behavior in VR, such as the influence of a student’s sitting position, the behavior of peer learners, and the visual representation of visual attention in a virtual classroom environment [30,33]. Both studies investigated participants at the ages of 10–13 years. Other researchers focused on medical applications or the classification and training of cognitively impaired participants [34]. By using different hardware, they were able to investigate the age group of 6–13 years. In the last few years, several groups have made an effort in reviewing the studies employing virtual reality for the examination of children [35,36,37,38]. Since children belong to a vulnerable group according to the Declaration of Helsinki [39] and the results of the previous 2D experiment suggested similar trends for children and adults in conditions without noise, adult participants were investigated to gain first insights into the feasibility of investigating ASA in VR using this specific paradigm.

### 1.5. Virtual Reality Questionnaires

There are numerous measures to evaluate a virtual environment and the participants’ immersion in this environment. Two very popular questionnaires are the Simulator Sickness Questionnaire (SSQ) [40] and the iGroup Presence Questionnaire (IPQ) [41].

The SSQ was first introduced by Kennedy in 1993 for the quantification of simulator sickness and used data from navy simulators to develop a total of 16 items in the categories nausea, oculomotor, and disorientation [40]. German translations of the items were suggested by Hosch [42]. Usually, the SSQ is rated before and after an experimental condition to establish a baseline and the effect of the virtual environment. Each item is rated on a four-point Likert scale ranging from 0 (not at all) to 3 (strong). For the evaluation, all items are weighted to receive scores for each category, as well as summed to obtain an overall score according to a formula derived by [40]. Since the questionnaire was proposed nearly 30 years ago, several studies have validated the items using newer technologies, e.g., [43,44]. While the use of the SSQ is still widely accepted to evaluate VR instead of simulators, there are some drawbacks to consider when using the SSQ and the proposed evaluation schema. When interpreting the results, it is important to keep in mind that there are no norm scores, although total scores above 20 are considered to indicate simulator sickness. However, these values have been developed based on military aviators, which might not reflect the general population directly [44].

The IPQ was developed for the quantification of presence and contains 14 items, which belong to the categories general presence (G), spatial presence (SP), involvement (INV), and experienced realism (REAL). The items have been developed either directly for the IPQ or taken from previous studies. The questionnaire is available in English, German, Dutch, French, and Japanese. Each question uses unique anchors, which are rated on a seven-point scale. One example is a question on the perceived realism “How real did the virtual world seem to you?”, which is rated from ”completely real” to “not real at all” [41].

### 1.6. Objectives

The present study aimed to develop a new paradigm for the investigation of the intentional switch of auditory selective attention in a VR classroom scenario. To explore the feasibility of such a study in VR and to validate the new method, a listening experiment was conducted, and the results are compared with the previous 2D version of this paradigm by Loh et al. [18]. Parts of this study were previously presented at the 47th Annual Conference on Acoustics DAGA 2021 in Vienna [45]. It is hypothesized that the VR paradigm used in this study will yield the same main effects as the 2D version since the basic structure of the paradigm has not changed. However, in accordance with previous studies that found evidence for enhanced attention in VR, e.g., [26], it is further hypothesized that auditory attention is increased in the VR environment, resulting in lower reaction times and error rates.

## 2. Materials and Methods

### 2.1. Participants

Following previous studies using similar paradigms on the voluntary auditory selective attention switch [9,10,11,12,13,18,46], 24 adults (age: 21–36 years, *M* = 27 years, SD = 4.04 years, 9 female) were recruited for the VR experiment, of which three had to be excluded after the screening (final group: age: 21–35 years, *M* = 26.8 years, SD = 3.7 years, 8 female). Inclusion criteria for both experiments were German-speaking and normal hearing abilities (within 25dB[HL] [47]), which was tested before the experiment with an Auritec Ear 3.0 audiometer for frequencies between 250Hz and 8kHz using a pulsed pure tone ascending standard audiometry [48]. For the VR experiment, normal or corrected to normal vision acuity (20/30) was required, which was tested using a Snellen chart [49]. Normal color vision was tested using a subset of Ishihara color charts (charts: 1, 2, 4, 8, 10, 14, according to instructions for quick testing) [50]. All participants confirmed that they had never participated in a listening experiment on auditory selective attention before and gave informed consent before the experiment. In the following, this group of participants will be referred to as the “VR group”.

The results of the new VR study were compared to the previous 2D audio-only study reported by Loh et al. [18]. Only the data obtained from the adult participants in conditions without background noise were further analyzed. In the previous experiment, 24 adults (age: 18–26 years, *M* = 22 years, SD = 2 years, 12 female) participated [18]. In the following, this group of participants will be referred to as the “2D group”.

### 2.2. Stimulus Material

The stimulus material consisted of eight animal names in the German language, which were recorded in an anechoic chamber at the Institute for Hearing Technology and Acoustics using a Neumann TLM 170 condenser microphone with a Zoom H6 recording device, which allows for recordings with a frequency range of 70Hz to 20kHz at a sampling rate of 44.1kHz and a quantization of 24bit. The voices of an adult female (24 years) and a male child (5 years) were recorded. The animal names are categorized into flying animals (“Biene” (bee), “Ente” (duck), “Eule” (owl), “Taube” (dove)), and non-flying animals (“Schlange” (snake), “Ratte” (rat), “Robbe” (seal), “Katze” (cat)) [18]. All stimuli were time-stretched or shortened while keeping the original frequency distribution to a length of 600ms using the “change tempo” algorithm provided by the open-source software Audacity [51]. All stimuli were loudness-normalized according to EBU-R128 [52].

### 2.3. Auditory Reproduction

Both experiments used a binaural synthesis that was reproduced using the software Virtual Acoustics (VA), [53], in the audio-only case using VA version 2018b with the integration for MATLAB and in the VR case using VA version 2021 and the Unity plugin. In both cases, a generic head-related transfer function (HRTF) of the IHTA artificial head with a resolution of $5^{\circ }\times 5^{\circ }$ was used [54]. For the audio-only version, the HRTFs were individualized by modifying the interaural time difference cues based on the participant’s head dimensions [55]. The audio-only version used a static reproduction, whereas the VR version used a dynamic reproduction. However, previous observations during the audio-only experiments showed that the participants rarely moved their heads. Further, investigations on a previous version of the paradigm between static and dynamic reproduction showed no significant differences [56]. The stimuli were played over open headphones (Sennheiser HD650). A perceptually robust headphone equalization was used for the experiments [57]. Therefore, headphone transfer functions (HpTFs) were measured for each participant using Sennheiser KE3 microphones at the blocked ear canal entrance and sweeps. The measurements were repeated six times, and the participants were asked to readjust the headphones after each measurement. The final filter was calculated as a minimum-phase filter using the average of the measurements. The HpTFs were measured and calculated using the ITAtoolbox for Matlab [58]. The Matlab code, as well as the respective version of the ITAtoolbox are available on Zenodo [59].

### 2.4. Visual Reproduction

**Figure 1:**
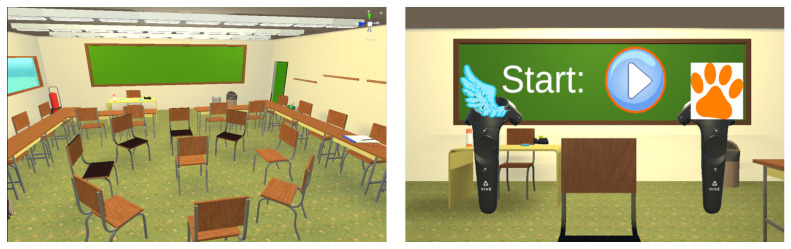
Images of the virtual environment. **Left**: Virtual classroom with the participant’s chair surrounded by a circle of chairs and basic classroom furniture. **Right**: View from the participant’s position in the virtual environment facing the blackboard and the virtual controller models.

A virtual classroom model with furniture was created using SketchUp make 2016 [60] to allow for room acoustic simulations using RAVEN [61] in the future. A further refinement, such as adding an outdoor environment, was made using the Unity software with the game engine version 2019.4.21f [62]. The experimental paradigm was implemented and executed in the Unity software. During the experiment, the participant was placed in the center of the classroom surrounded by a circle of chairs representing the possible stimulus positions (Figure 1 (left)). The participant faced a blackboard on which all instructions, the visual cue, and feedback were presented. Figure 1 (right) shows the participant’s view in the virtual classroom at the beginning of the experiment. The Unity project is available on Zenodo [59].

For visual presentation, an HTC Vive Pro Eye head-mounted display (HMD) was used. Before the experiment, participants were asked to adjust the HMD according to their head dimensions until they could see sharply. The corresponding HTC Vive controllers served as the input devices, which were represented by virtual models of the controllers in the VR environment (see Figure 1 (right)). During the instruction phase, laser beams were emitted from the virtual controller models to facilitate the selection of buttons displayed on the blackboard. For the experiment phase, the laser beams were hidden and images of a wing and paw were displayed on the virtual controllers, indicating which controller corresponded to which answer option. For input selection, the participants used their index fingers to press down the trigger on the bottom of the controllers. To achieve a smooth visual presentation of the virtual environment, a frame rate of 90fps was ensured during the whole experiment.

### 2.5. Experiment Room and Virtual Classroom Setup

The experiment was conducted in an acoustically treated hearing booth ($l\;\times \;w\;\times \;h=2.3\;\times \;2.3\;\times \;1.98\;m^{3}$) at the Institute for Hearing Technology and Acoustics, RWTH Aachen University, to ensure a quiet environment.

The dimensions of the virtual classroom had to be larger compared to a real-life classroom ($l\;\times \;w\;\times \;h=10\;\times \;9\;\times \;3\;m^{3}$) to fit the circle of chairs with a diameter of 2 m, which represented the stimulus positions. The large classroom allowed for the placement of a big blackboard to ensure good visibility of the instructions. Next to sparse furniture and decoration, the left side of the classroom was fit with big windows (see Figure 1).

### 2.6. Evaluation of the Virtual Environment

To have an impression of the interaction with the virtual environment, the position and rotation data of the HMD were tracked during each trial.

Further, the SSQ was rated by each participant before and after the experiment while being seated in the virtual world. The IPQ was only rated after the experiment. Both questionnaires were displayed on the virtual blackboard, and each point of the scale was represented by a button.

### 2.7. Experimental Procedure

During the experiment, the participants were seated in a virtual classroom, which was displayed on an HTC Vive Pro Eye head-mounted display. HTC Vive Pro Eye controllers were used to interact with the virtual world. The experiment consisted of a visual and auditory screening, a short training phase, and the experimental blocks.

**Figure 2:**
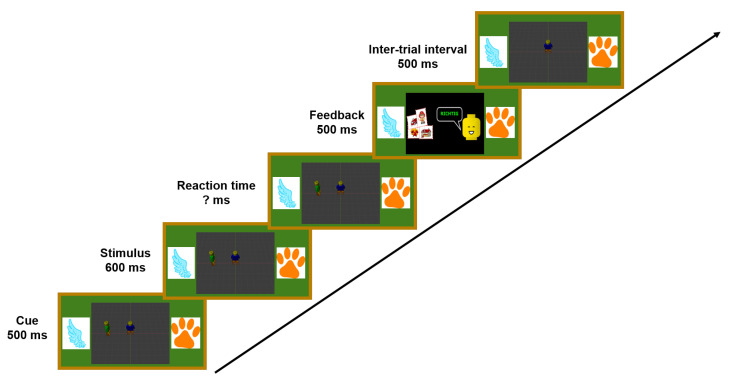
Schematic of the trial structure including the instructions on the virtual blackboard and all experiment intervals.

**Figure 3:**
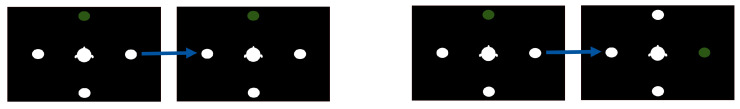
Transition of the target position between trials causing a switch of the auditory attention. Left: Repetition of the target position between trials. Right: Switching of the target position from front to right between trials.

The main task in the paradigm was to categorize a previously cued auditory target stimulus. During each trial of the experiment, two auditory stimuli were presented simultaneously, one target and one distractor. In each trial, they could either belong to the same category or different ones. Figure 2 gives an overview of one trial within the experiment. At the beginning of each trial, the target position was cued using a visual representation on a virtual blackboard situated in front of the participant. The stimuli could be presented from one of four positions around the participant (front, back, left, or right), while the target and distractor were never at the same position during one trial (see, e.g., Figure 3). The visual cue was displayed for 500ms. Afterward, the stimuli were played back from the respective position, and the participants gave their answers using the controllers by pressing the trigger button. There was no limitation to the reaction time. Before the experiment, participants were told which controller belonged to which category.

After the participant’s answer, visual feedback was presented on the blackboard showing a positive or negative smiley. The feedback was displayed for 500ms. Afterward, the inter-trial interval of 500ms began, which was followed by the next cue. Between each trial, the distractor position was changed, while the target position could be repeated or changed. Next to the feedback after each trial, three to five stars were shown after each block to represent the success rate during the block. Additionally, the progress during the experiments was indicated to keep the participant motivated. The participant could choose freely when to continue with the next experiment block. The total experiment duration was about 60min including 20–30 min of preparations.

### 2.8. Experimental Design

Four independent variables were evaluated. The experiment group (GR) with the levels 2D group and VR group was evaluated as a between variable. All other independent variables were investigated within subjects: the attention transition (AT) with the levels switch and repetition, the congruency of target and distractor stimulus (C) with the levels congruent and incongruent, as well as the combination of target and distractor position (TD-PC) with the levels left–right (LR), next-to (NEXT) and front–back (FB). The dependent variables reaction time (RT) and error rates (ER) were evaluated for all conditions. In the VR group, each test condition was repeated 16 times. This led to a total number of 192 experiment trials excluding the training.

#### 2.8.1. Experiment Group

Two groups were evaluated in this study, the adult participants of a previous auditory-only study and the participants of the current VR experiment. Further information on the participants for this study is given in Section 2.1. Information on the participants of the previous audio-only study is given in Loh et al. [18].

#### 2.8.2. Attention Transition

As indicated in Figure 3, the position of the target stimulus was manipulated between trials, introducing attention switches in half of the trials. The target stimulus’ position could either stay the same (repetition, e.g., front–front, top part of Figure 3) or change (attention switch, e.g., front-right, bottom part of Figure 3). By changing the target position, the auditory attention needed to be refocused. The distractor’s position was changed between all trials.

#### 2.8.3. Congruency

**Figure 4:**
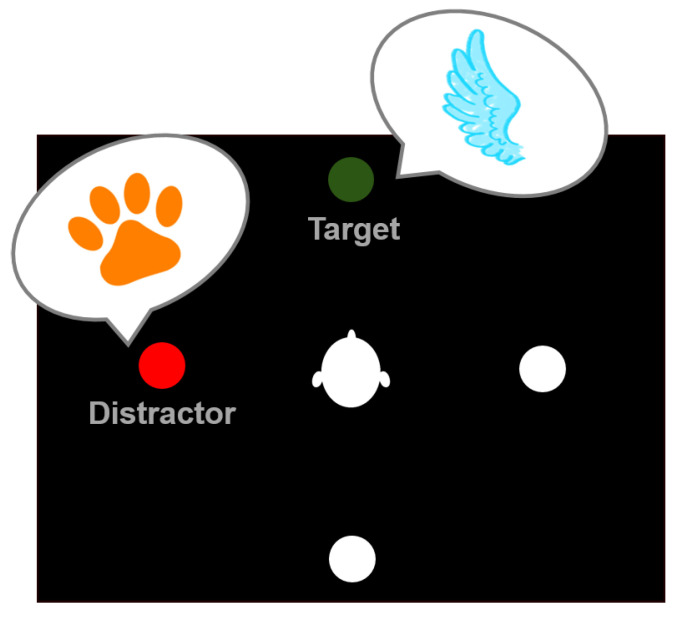
Exemplary trial indicating the target stimulus at the frontal and the distractor at the left position. The target stimulus belongs to the category of flying animals, while the distractor is a non-flying animal, yielding an incongruent trial.

The variable congruency refers to the stimuli’s content in each trial. During a congruent trial, the target and distractor belonged to the same category (e.g., both flying or both non-flying). In incongruent trials, both stimuli presented names from different categories as indicated in Figure 4 (e.g., target: flying and distractor: non-flying). Congruent trials were expected to be easier to answer and require less cognitive load.

#### 2.8.4. Target–Distractor Position Combination

**Figure 5:**
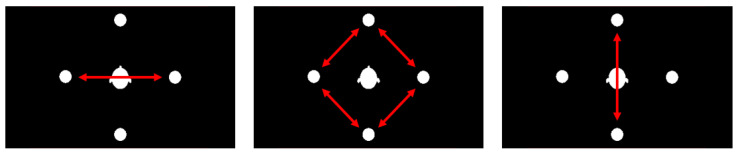
Target–distractor position combinations. **Left**: left–right; **center**: next-to; **right**: front–back.

Due to the four chosen stimulus positions (front, back, left, right), the combination of the target–distractor position had three levels, as indicated in Figure 5: left–right, next-to (e.g., front-left or right-back), and front–back. As a binaural synthesis with a non-individualized HRTF used for acoustic playback, left–right trials were expected to yield the lowest error rates and reaction times due to the spatial separation of the sound sources, while front–back trials were expected to yield the worst performance due to in-head localization and front–back confusions.

## 3. Results

This section compares the findings of the audio-only 2D paradigm and the new VR version.

For every trial, reaction times (RTs) in milliseconds (ms) and error rates (ERs) (% false) were measured and analyzed. Training trials, the first trial of each block, and trials following a false answer were removed from the data. For the RTs, a Z-transformation was applied for each participant, allowing for the removal of trials exceeding ±2 standard deviations as outliers (4.8%). For RT analysis, also error trials were removed.

To investigate whether the independent variables had an influence on the reaction times and error rates and to compare the different conditions, repeated-measures analyses of variance (ANOVAs) [63,64] with the between-subjects variable of the experiment group and the within-subjects variables attention transition, congruency, and target–distractor position combination (see Section 2.8) were performed separately for ERs and RTs to investigate the main and interaction effects. If Mauchly’s test indicated a violation of the sphericity assumption, the degrees of freedom were corrected using Greenhouse–Geisser estimates. For further insight, post hoc tests with Bonferroni correction were conducted if the ANOVA revealed effects. An overview of all ANOVA results is given in Table 1. Therefore, only significant main and interaction effects, as well as relevant post hoc analyses are stated explicitly.

### 3.1. Reaction Times

**Figure 6:**
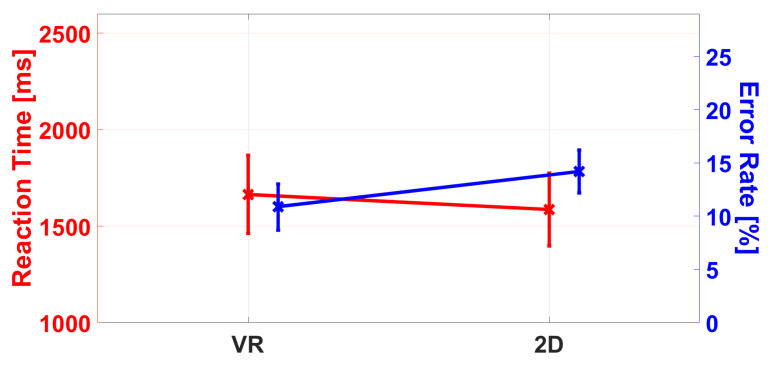
Differencein reaction times (RT, red in ms) and error rates (ER, blue in %) between the 2D and VR groups. In the reaction time, a trend toward lower RTs in the 2D group can be observed, which is not significant. The ANOVA revealed a significant effect in ERs, indicating higher ERs in the 2D group. The error bars represent the standard error.

The main effect of GR was not significant, $F\left(1,43\right)=0.323,p=0.573,\eta_{p}^{2}=0.007$, indicating comparable RTs for the VR and 2D groups (1664.47 ms vs. 1586.35 ms) (see Figure 6).

The ANOVA revealed a main effect of AT, $F\left(1,43\right)=15.762,p<0.001,\eta_{p}^{2}=0.268$, indicating lower RTs for repetition trials than for switch trials (1586 ms vs. 1665 ms). A main effect of C, $F\left(1,43\right)=32.016,p<0.001,\eta_{p}^{2}=0.427$, suggests lower RTs in congruent trials compared to congruent trials (1559 ms vs. 1692 ms). Mauchly’s test indicates that the assumption of sphericity was violated for TD-PC ($\chi^{2}=61.674,p<0.001$). Therefore, the degrees of freedom were corrected using Greenhouse–Geisser estimates ($\epsilon_{gg}=0.565$). The main effect of TD-PC was significant, $F\left(1.1,48.6\right)=44.942,p<0.001,\eta_{p}^{2}=0.682$. Bonferroni-adjusted post hoc tests revealed significantly lower RTs for LR (1365 ms) vs. next-to (1616 ms) conditions (p<0.001), as well as for FB (1895 ms) conditions (p<0.001). The comparison of next-to and FB was also significant (p<0.001).

As Mauchly’s test indicated that the assumption of sphericity was violated for the interaction of AT × TD-PC ($\chi^{2}=16.105,p<0.001$), the degrees of freedom were corrected using Greenhouse–Geisser estimates ($\epsilon_{gg}=0.758$). The interaction was not significant, $F\left(1.5,65.2\right)=3.104,p=0.065,\eta_{p}^{2}=0.067$. The differences between the mean values indicate a tendency towards stronger TD-PC (LR vs. next-to vs. FB) effects in repetition trials than in switch trials (1376 ms vs. 1660 ms vs. 1958 ms and 1354 ms vs. 1573 ms vs. 1832 ms).

**Figure 7:**
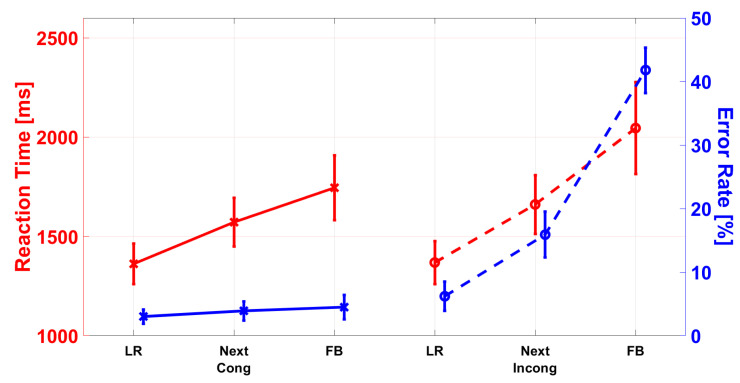
Difference in reaction times (RT, red in ms) and error rates (ER, blue in %) between congruent (cong) and incongruent (incong) stimuli for the target–distractor position combination (TD-PC). In the reaction times, the ANOVA revealed an interaction effect, indicating lower RTs in congruent than incongruent trials for next-to and FB conditions. In the error rates, significantly lower ERs were found for congruent than incongruent trials in LR, next-to, and FB conditions. The error bars represent the standard error.

For the interaction of C × TD-PC, Mauchly’s test indicated that the assumption of sphericity was violated ($\chi^{2}=8.246,p=0.016$). Therefore, the degrees of freedom were corrected using Greenhouse–Geisser estimates ($\epsilon_{gg}=0.849$). The interaction was significant, $F\left(1.7,73.0\right)=23.014,p<0.001,\eta_{p}^{2}=0.349$ (see Figure 7). Bonferroni-adjusted post hoc tests showed lower RTs in congruent than incongruent trials for next-to conditions (p=0.005, 1572 ms vs. 1661 ms) and FB conditions (p<0.001, 1744 ms vs. 2045 ms). The comparison of LR trials was not significant (p>0.05).

All other two-way interactions showed no significant effects.

None of the three-way interactions showed significant effects.

### 3.2. Error Rates

The ANOVA revealed a main effect of GR, $F\left(1,43\right)=5.259,p=0.027,\eta_{p}^{2}=0.109$, indicating lower ERs in the VR group than in the audio-only group (10.9% vs. 14.2%; see Figure 6).

The main effect of C was significant, $F\left(1,43\right)=263.320,p<0.001,\eta_{p}^{2}=0.860$, indicating lower ERs for congruent than for incongruent trials (3.8% vs. 21.3%).

Mauchly’s test indicated that the assumption of sphericity was violated for TD-PC ($\chi^{2}=17.098,p<0.001$). Therefore, the degrees of freedom were corrected using Greenhouse–Geisser estimates ($\epsilon_{gg}=0.749$). The main effect of TD-PC was significant, F(2,42)=125.094,$p<0.001,\eta_{p}^{2}=0.856$. Bonferroni-adjusted post hoc tests indicated lower ERs for FB than next-to and FB conditions (p<0.001, 4.6% vs. 9.9% vs. 23.2%). The difference between next-to and FB was also significant (p<0.001).

**Figure 8:**
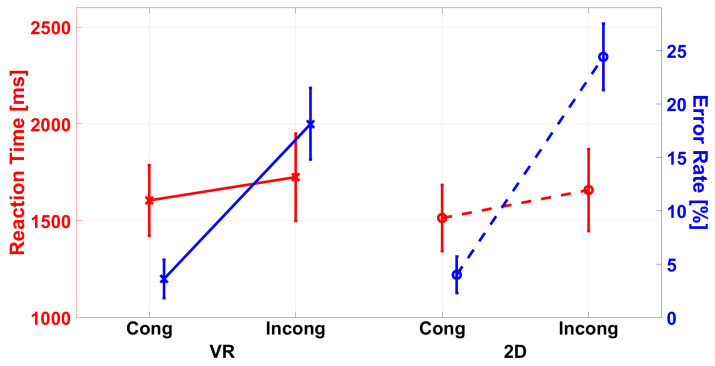
Difference in reaction times (RT, red in ms) and error rates (ER, blue in %) between the 2D and VR group for the congruency. In the reaction time and error rates, the ANOVA revealed a main effect of congruency, indicating lower RTs and ERs in congruent trials. For ERs, the ANOVA also revealed an interaction with the group indicating lower ERs for the VR than the 2D group in incongruent trials. The error bars represent the standard error.

The interaction of C × GR was significant, $F\left(1,43\right)=7.492,p=0.009,\eta_{p}^{2}=0.148$ (Figure 8). Bonferroni-adjusted post hoc tests revealed lower ERs in incongruent trials for the VR group than the audio-only group (p=0.008, 18.1% vs. 24.4%). The differences in congruent trials were not significant.

The interaction of C × TD-PC was significant, $F\left(2,86\right)=170.319,p<0.001,\eta_{p}^{2}=0.798$. Bonferroni-adjusted post hoc tests showed significantly lower ERs for congruent than incongruent trials in LR (p=0.010, 3.2%), next-to (p<0.001, 11.9%), and FB (p<0.001, 37.2%) conditions, as indicated in Figure 7.

All other two-way interactions showed no significant effects.

None of the three-way interactions showed significant effects.

### 3.3. Head Movement

For the VR version, head movement was tracked by saving the position and rotation of the HMD with a correction for the head center. The positions were normalized for every participant separately concerning the participant’s head position at the beginning of the experiment. A brief evaluation of the results revealed that no participant rotated the head more than 5°. Due to the lack of movement, no detailed evaluation of the head rotation was conducted.

### 3.4. Questionnaires

The results obtained for the SSQ were calculated according to Kennedy [40]. The total SSQ scores before and after the experiment were $M_{\mathrm{total},\mathrm{start}}=35.8$ and $M_{\mathrm{total},\mathrm{end}}=48.6$. To underline the effect caused by the virtual environment, the results before the experiment were subtracted from the results after the experiment for each participant, and a difference score was calculated to be $M_{\mathrm{total},\mathrm{difference}}=12.8$. Table 2 gives the mean values for the subscales, as well as the standard deviation, the minimum, and the maximum values for each measure [44].

For the IPQ, ratings were given on a scale from 0 to 6. Attributes with inverse scales were corrected according to the IPQ guidelines [41]. A total mean score of $M_{G}=3.43$ was achieved for the general presence. For spatial presence, involvement, and experienced realism, the ratings were $M_{\mathrm{SP}}=3.88$, $M_{\mathrm{INV}}=3.79$, and $M_{\mathrm{REAL}}=2.15$, respectively. Table 3 shows additional standard deviations, as well as minimum and maximum values.

## 4. Discussion

This study aimed to translate a paradigm on the intentional switching of auditory selective attention into virtual reality and validate it using a listening experiment. The basic paradigm structure was previously developed by Loh et al. [18]. It was hypothesized that the main effects would show the same tendencies in the 2D and VR versions. Additionally, an increase in performance due to enhanced attention in VR was expected.

While the error rates can be directly interpreted, the absolute values for the reaction times should be put into context, since they are strongly dependent on the task and measurement method. Previous studies found that the auditory reaction time is in the range of 210–285 ms when responding to the onset of a pure tone [65,66,67]. Further, the processing time increases with the complexity of the task, e.g., the semantic processing starts 200–300 ms after a stimulus is played [68]. In the classification of numbers, numbers that are numerically further apart can be classified more easily than numbers that are closer together. For example, the numbers “2” and “3” are distinguished more slowly than the numbers “2” and “7”, due to their numerical distance [69]. Since the task presented in the current study is more complex, the reaction times could be expected to be higher. In the presented study, significant differences in reaction times started from 100 ms for the main effect of attention transition, yielding a difference of about 5%. A larger difference was found for the target–distractor position combination, where the mean reaction times difference was 530 ms or 38% in the left–right vs. front–back condition. Therefore, the differences were in the time range of the auditory reaction time, as well as semantic processing. Overall, the reported reaction times were in the same range as previous studies (1000–2500 ms) [12,13,18].

All in all, the main effects, as well as the interaction effects yielded the expected results and, most importantly, showed the same tendencies in the 2D and the VR version of the paradigm, as well as previous studies employing the same paradigm in an adult version [11,18]. This reflects the good reproducibility, robustness, and validity of the overall paradigm.

### 4.1. Group Differences

The results of the present study showed the same tendencies as previous work [22,26] since the error rates were significantly lower in the VR group. The interaction effect of group and congruency further suggests that the performance was only better in incongruent trials. However, this VR advantage was not visible in the reaction times. These results could be due to enhanced attention in VR, as proposed by Li et al. [26]. Verbal feedback by the participants also suggests that they felt immersed in the VR environment, which might have introduced an extended focus [70]. Furthermore, the overall gamification can lead to an enhanced engagement in the task [29]. However, another reason could be that the VR experiment overall was shorter than the 2D version, since the 2D experiment also contained background noise conditions, which are more demanding. This could have led the participants to try to finish early and take the risk of false responses.

### 4.2. Attention Transition

The auditory attention transition was expected to yield switch costs in trials where the target position was changed and the attention needed to be refocused. In accordance with previous studies (e.g., [9,10,11,18]), the results indicate that the spatial reorientation of one’s attention was more demanding than staying focused on one target direction, which was indicated by longer reaction times in the switch conditions for all participants. This effect was not reflected in the error rates.

### 4.3. Congruency

The congruency of target and distractor stimuli was expected to facilitate the task as opposed to incongruent trials. The results supported this by showing significantly higher error rates and longer reaction times in incongruent trials. In incongruent trials, the VR group achieved significantly lower error rates, which could indicate that the information processing is facilitated in the VR condition and that the distractor could more easily be suppressed.

### 4.4. Target–Distractor Position Combination

As a third independent variable, the combination of the target–distractor position was investigated. This represented the spatial separation of the sound sources. Due to the use of a generic HRTF, it was expected that the left–right conditions were best and the front–back conditions were worst in being distinguishable. In accordance with previous studies, the results support this assumption (e.g., [9,10,11,18]). The trend in TD-PC can be explained by the best source separation in the LR condition followed by the next-to condition, whereas the FB cases led to in-head localization and front–back confusions.

Next to the visual setting, the 2D version used a static reproduction with an individualized HRTF with an ITD adjustment, whereas in the VR version, a dynamic reproduction with a generic HRTF was used. According to Oberem et al. [71], who investigated the differences of the same reproduction system, front–back confusions and in-head localizations were expected to decrease in a dynamic reproduction. Since no interaction effect of the group and target–distractor position combination was found, also no difference between the individualized static and generic dynamic reproductions can be derived from this study. Contradictory results regarding the benefit of dynamic and individualized HRTFs were previously reported by Oberem et al. [56] when comparing a static and dynamic reproduction with a previous version of this paradigm. They found higher error rates in the dynamic reproduction and lower reaction times in the dynamic one. While these results are highly inconclusive, one reason could be the lack of head movements. In both studies, the head movements were in the range below 5°.

### 4.5. Virtual Environment

The high overall sores in the SSQ after the experiment suggest that the virtual environment caused discomfort. After the experiment, all subscales of the SSQ were rated higher than the suggested norm value of 20. The highest scores were given to the attributes related to disorientation before, as well as after the experiment. Since primarily the attributes “blurred vision” and “dizziness with eyes open” were rated poorly, the high sickness scores could be attributed to a bad rendering of the written questions, as well as an incorrect adjustment of the HMD. In future experiments, the participants should get a better introduction on how to put on the HMD correctly and adjust it according to the individual head dimensions to ensure the sharpness of vision. It should also be noted that the reported SSQ scores were collected in the virtual environment, whereas usually, the questionnaires are answered in the real world. Therefore, participants had no chance to regenerate from any impairments caused by the virtual scene or the equipment. However, for other questionnaires, the results have been shown to be comparable when collecting the data in the real as opposed to the virtual world [72].

The IPQ scores for general and spatial presence, as well as involvement indicate that the participants had a very light tendency towards feeling present in the virtual world. However, the scores for the experienced realism indicate that the virtual environment appeared to be artificial. The lack of presence can be explained by the overall visualization, which was rather rudimentary and did not aim to be highly realistic. Furthermore, the enhanced interaction with the virtual environment might increase the sense of being integrated into the scene.

### 4.6. Limitations and Future Directions

#### 4.6.1. Participant Group

Next to physiological restrictions, such as children’s head dimensions, there are age restrictions by many HMD manufacturers. In the last few years, also ethical concerns have been raised since children might be more susceptible to game-like elements and the virtual world. However, these ethical discussions remain unsolved regarding children, as well as adults as the target group (see, e.g., [70,73,74] for reviews). Therefore, to validate the feasibility of the new VR paradigm, adult participants were investigated instead of children, which belong to a vulnerable participant group. In future studies, it would be very interesting to see if the decrease in error rates in the VR environment is reflected in children and how this interacts with their susceptibility to noise. These studies should take care to create a child-appropriate VR environment. Measures to ensure a safe experience could be an introduction to the virtual world, monitoring during the experiment as well as after finishing the experiment to make sure that the children can distinguish between the real and virtual environments [75].

#### 4.6.2. Auditory Reproduction

The benefits of the dynamic binaural reproduction, which usually improves front–back confusions and in-head localization drastically [71], are rarely exploited due to the paradigm design. Since all the instructions, the cue, and the feedback were presented on a display in the 2D version and a blackboard in the VR classroom, which was positioned in front of the participant, the presented task was very static. This motivated very little head movement. Furthermore, previous studies found the same effect that, in this particular paradigm, a dynamic reproduction did not yield the expected benefit since participants did not move [11]. In a future version of this paradigm, the actual behavior in a real-life classroom could be considered. For example, the use of different cueing methods could motivate the participants to move their heads.

#### 4.6.3. Visual Reproduction

Compared to other studies (e.g., [30,33,34]), the virtual classroom presented here is not very realistic due to unusual room dimensions, cartoonish textures, and sparse furniture. This might decrease the level of immersion in the virtual world. To increase the realism of the classroom scenario, a more realistic room model should be created. Further, no visual representations of the sound sources, e.g., loudspeakers or virtual agents, were placed in the room, which is not realistic. For further studies, the integration of virtual peers should be considered to increase engagement in the scene [30].

#### 4.6.4. Tracking System

It is well known that the in-built Vive tracking system is not as accurate as other commercial systems, especially if movements are involved [76]. If future adaptations of the VR paradigm encourage the participants to move and interact with the virtual environment, the use of a more accurate tracking system, such as the OptiTrack Motion Capture System, should be evaluated [77].

## 5. Conclusions

This study presented a VR paradigm to measure the intentional switching of auditory selective attention. As hypothesized, the same main effects as in the previous 2D version were found in the VR version of the paradigm, which not only underlines the robustness of the paradigm, but also confirms the validity of the newly introduced VR version. However, the decrease in the error rates for the VR group also suggests that the participants’ attention is enhanced in the VR scenario.

Since this experiment was intended to investigate the auditory attention switch of children in a more realistic environment, further steps need to be taken to improve the realism, e.g., by adding noise, upgrading the visual representation, adding agents, or creating a school lecture scenario. However, there are ongoing ethical discussions regarding the influence of VR on children, which need to be evaluated before investigating child participants.

## Figures and Tables

**Table 1:** Repeated-measures ANOVA results for reaction time and error rate.

Within-Group Variable	Reaction Time				Error Rate			
	df	F	p	$\eta_{p}^{2}$	df	F	p	$\eta_{p}^{2}$
GR	(1,43)	0.323	0.573	0.007	**(1,43)**	**5.259**	**0.027**	**0.109**
AT	**(1,43)**	**15.762**	**<0.001**	**0.268**	(1,43)	0.037	0.848	0.001
C	**(1,43)**	**32.016**	**<0.001**	**0.427**	**(1,43)**	**263.320**	**<0.001**	**0.860**
TD-PC	**(1.1,48.6)** ${}^{a}$	**44.942**	**<0.001**	**0.682**	**(1.5,64.4)** ${}^{a}$	**142.817**	**<0.001**	**0.769**
AT × GR	(1,43)	2.088	0.156	0.046	(1,43)	0.026	0.873	0.001
AT × C	(1,43)	1.501	0.227	0.034	(1,43)	0.677	0.415	0.015
AT × TD-PC	(1.5,65.2) ${}^{a}$	3.104	0.065	0.067	(1.7,74.6) ${}^{a}$	1.227	0.295	0.028
C × GR	(1,43)	0.279	0.600	0.006	**(1,43)**	**7.492**	**0.009**	**0.148**
C × TD-PC	**(1.7,73.0)** ${}^{a}$	**23.014**	**<0.001**	**0.349**	**(2,86)**	**170.319**	**<0.001**	**0.798**
TD-PC × GR	(2,86)	2.135	0.124	0.047	(2,86)	1.804	0.171	0.040
AT × C × GR	(1,43)	1.293	0.262	0.029	(1,43)	0.016	0.898	<0.001
AT × TD-PC × GR	(2,86)	0.254	0.776	0.006	(2,86)	0.733	0.483	0.017
C × TD-PC × GR	(2,86)	0.120	0.887	0.003	(2,86)	1.917	0.153	0.043
AT × C × TD-PC	(1.5,65.6) ${}^{a}$	2.306	0.120	0.051	(1.7,71.3) ${}^{a}$	0.775	0.443	0.018
AT × C × TD-PC × GR	(2,86)	0.269	0.764	0.006	(2,86)	0.306	0.737	0.007

*Notes:* GR = experiment group; AT = attention transition; C = congruency; TD–PC = target–distractor position combination. Significant effects are indicated in bold. ^a^ Greenhouse–Geisser correction applied due to violation of sphericity assumption.

**Table 2:** Simulator sickness results.

		Mean	SD	Min	Max
Start					
	Nausea	11.4	16.8	0.00	66.8
	Oculomotor	28.2	16.2	0.00	68.2
	Disorientation	65.0	39.9	13.9	181.0
	Total	35.8	21.3	3.70	86.0
End					
	Nausea	20.9	19.7	0.00	76.3
	Oculomotor	34.7	20.7	7.58	75.8
	Disorientation	86.8	49.3	13.9	194.9
	Total	48.6	27.5	7.48	108.5
Difference (End-Start)					
	Nausea	9.54	11.8	−9.54	38.1
	Oculomotor	6.50	13.1	−15.2	45.5
	Disorientation	21.9	34.3	−41.8	111.4
	Total	12.8	18.0	−18.7	59.8

**Table 3:** IPQ results.

	Mean	SD	Min	Max
G	3.43	1.43	0	6
SP	3.88	1.01	1.8	5.4
INV	3.79	1.28	1.5	5.75
REAL	2.15	0.71	1	3.5

## Data Availability

The software used to conduct the presented study, as well as the collected data are publicly available in “Auditory Selective Attention Switch in a Virtual Reality Classroom Environment” at https://doi.org/10.5281/zenodo.7248833 under the Creative Commons Attribution 4.0 International license. The auditory stimuli are not included in this dataset, but are available upon request from the corresponding author.

## Abbreviations

The following abbreviations are used in this manuscript:

ANOVA	Analysis of variance
ASA	Auditory selective attention
AT	Attention transition
C	Congruency
CTC	Cross-talk cancellation
ER	Error rate
FB	Front–back condition
G	General presence
GR	Experiment group
HMD	Head-mounted display
HpTF	Headphone transfer function
HRTF	Head-related transfer function
IHTA	Institute for Hearing Technology and Acoustics, RWTH Aachen University
INV	Involvement
IPQ	iGroup Presence Questionnaire
LR	Left–right condition
Next	Next-to condition
REAL	Experienced realism
RT	Reaction time
SP	Spatial presence
SSQ	Simulator Sickness Questionnaire
TD-PC	Target–distractor position combination
VA	Virtual acoustics
VR	Virtual reality
